# Spoligologos: A Bioinformatic Approach to Displaying and Analyzing *Mycobacterium tuberculosis* Data

**DOI:** 10.3201/eid0811.020174

**Published:** 2002-11

**Authors:** Jeffrey R. Driscoll, Pablo J. Bifani, Barun Mathema, Michael A. McGarry, Genét M. Zickas, Barry N. Kreiswirth, Harry W. Taber

**Affiliations:** * New York State Department of Health, Albany, New York, USA; † Institut Pasteur de Lille, Lille, France; and ‡Public Health Research Institute, New York, New York, USA

**Keywords:** tuberculosis, DNA fingerprinting, bioinformatics, spoligotyping

## Abstract

Spacer oligonucleotide (spoligotyping) analysis is a rapid polymerase chain reaction–based method of DNA fingerprinting the *Mycobacterium tuberculosis* complex. We examined spoligotype data using a bioinformatic tool (sequence logo analysis) to elucidate undisclosed phylogenetic relationships and gain insights into the global dissemination of strains of tuberculosis. Logo analysis of spoligotyping data provides a simple way to describe a fingerprint signature and may be useful in categorizing unique spoligotypes patterns as they are discovered. Large databases of DNA fingerprint information, such as those from the U.S. National Tuberculosis Genotyping and Surveillance Network and the European Concerted Action on Tuberculosis, contain information on thousands of strains from diverse regions. The description of related spoligotypes has depended on exhaustive listings of the individual spoligotyping patterns. Logo analysis may become another useful graphic method of visualizing and presenting spoligotyping clusters from these databases.

The process of DNA fingerprinting *Mycobacterium tuberculosis* isolates provides epidemiologists with data for investigating transmission and confirming laboratory cross-contamination. When personnel and resources are available, strains from all newly diagnosed cases of tuberculosis (TB) are fingerprinted. Interpreting the data from these sentinel surveillance studies can be challenging. TB can be latent for decades before resulting in active disease. The best method for examining *M. tuberculosis* complex genotyping data gathered over just a few years is still in question.

The genotyping of *M. tuberculosis* complex has been undertaken in the United States in directed studies and in sentinel surveillance. Since 1992, *M. tuberculosis* complex DNA fingerprinting for isolates has been available to U.S. Departments of Health and TB Control Offices to investigate cases of suspected TB transmission and suspected laboratory cross-contamination. At the same time, sentinel surveillance of select regions was used to evaluate if genotyping every new patient isolate from a particular region was useful. Although direct DNA fingerprinting studies are relatively simple to design, we are still learning how best to use in toto the large and diverse databases generated by sentinel surveillance studies from multiple laboratories (1–7). Previous studies have used DNA fingerprinting methods to understand the development and spread of subspecies of the *M. tuberculosis* complex, such as *Mycobacterium africanum* (8,9), *M. bovis* (10), and the W-Beijing family (11,12).

Analyzing the spread and evolution of *M. tuberculosis* complex strains is more complicated because of the long incubation of disease and relatively short-term collection of data (approximately 10 years). We examine whether bioinformatic tools can help in analyzing the data collected. Bioinformatics uses sophisticated analyses of large amounts of genetic information to clarify the relationships between species, explain the evolution of groups of genes, and assemble information from genome sequencing projects. Bioinformatic analysis involves searching nucleic acid or protein sequence information for previously unrecognized motifs that may signal previously unrecognized regions of interest. Spoligotype analysis (13) is a form of DNA sequencing by hybridization. Several groups have used some of these novel analytical tools to examine *M. tuberculosis* complex genotyping (4,6,14). Sequence logo analysis can find motifs in potentially related nucleic acid or protein sequences (15). Logo analysis combines these data on a graphic that illustrates the location and degree of sequence conservation in the selected sequences. We applied sequence logo analysis to find motifs based on the presence or absence of specific spacer sequences. We also evaluated the usefulness of logo analysis in examining phylogenetic relationships of the *M. tuberculosis* complex direct repeat (DR) (7) locus and its potential as a simple graphic method presenting grouped spoligotyping data.

We suggest using the sequence logo technique to understand the distribution of each the spacer sequences used in the spoligotype assay. This information is useful in improving or redesigning the spoligotype assay by showing the degree of differentiation achieved with each spacer sequence.

## Methods

### Isolates

Approximately 5,100 isolates of *M. tuberculosis* complex, predominately from patients in TB control programs in the northeast United States, are part of the Wadsworth Center spoligotype database. Most of these strains have been collected through ongoing sentinel surveillance projects in Massachusetts and New York City from 1996 to the present. In our strain collection, 920 different spoligotype patterns were identified.

### DNA Fingerprinting Analyses

Spoligotype analysis was performed according to a standard method (13). PCR amplifications were performed on extracted DNA or cell suspensions, which were heat-killed at 80°C for 1 hr in an oven. Spoligotype patterns were analyzed with BioImage Whole Band Analysis v3.4 software (Genomic Solutions, Ann Arbor, MI) on a Sun Ultra10 workstation (Sun Microsystems Inc., Santa Clara, CA). Spoligotype patterns were given descriptive nomenclature according to the standard method (16), along with a unique arbitrary numeric designation by the Centers for Disease Control and Prevention (CDC).

IS6110-based restriction fragment length polymorphism (RFLP) analysis was performed, according to standard protocol (17), at the Wadsworth Center (Albany, NY) and the Public Health Research Institute (New York City, NY), on approximately 3,700 of these isolates. RFLP patterns were analyzed with BioImage Whole Band Analysis v3.4 software (Genomic Solutions). RFLP pattern designations for sentinel surveillance isolates were assigned a unique arbitrary numerical designation by CDC.

### Spoligologo Analysis

Spoligologo denotes the application of sequence logo analysis to spoligotype assay data. Sequence logo analysis was originally devised as a method to find blocks of related amino acids between protein sequences and display the information in an intuitive visual description that illustrates both the residue and the degree of conservation at each position (18). Logo analysis has been used to look for functional and evolutionary relationships among groups of proteins and nucleic acids (19). Spoligologo analysis (15) was accomplished by using WebLogo software from the School of the Biological Sciences at the University of Cambridge (available from: URL: http://www.bio.cam.ac.uk/seqlogo/). To be compatible with WebLogo, letter designations (x=hybridization, o=no hybridization signal) were used to denote the pattern of hybridization observed for each spoligotype pattern. Spoligotype patterns to be compared were entered directly into WebLogo and a postscript file of the results was generated. Logo analysis compares each selected pattern against the other at the same position. Thus, we compared hybridization to spacer 1 in the group of selected spoligotypes, followed by analysis of spacer 2, and so on for all 43 spacers.

For convenience of illustration, two groups of isolates were chosen for spoligologo analysis. The first set consisted of 43 strains of *M. bovis* (13) in our collection. The second set was 12 low-band (exhibiting fewer than six copies of IS*6110* by RFLP analysis) *M. tuberculosis* isolates from Vietnam-born, Massachusetts sentinel surveillance case-patients. Vietnamese patients are the largest foreign-born group represented in the low-band data from Massachusetts.

## Results

**Figure 1:**
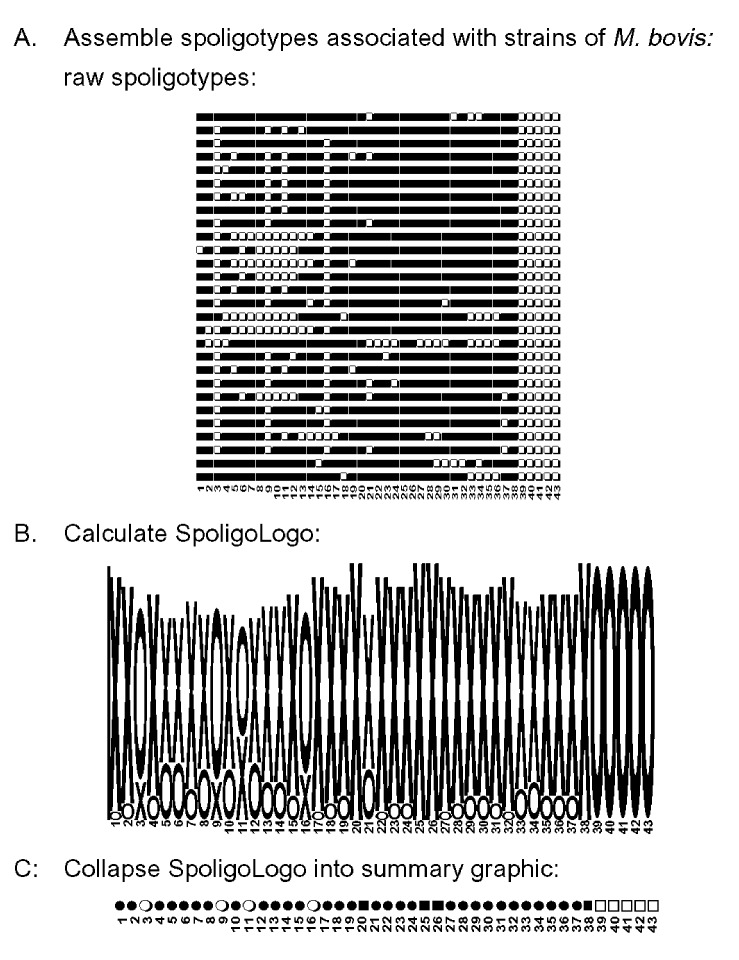
Logo analysis on spoligotypes associated with *Mycobacterium bovis.* The Wadsworth Center database contains 28 unique spoligotypes from strains of *M. bovis*. Panel A illustrates the raw hybridization data followed by the same patterns coded for logo analysis. To be compatible with WebLogo analysis, patterns were converted to a 43-character–long string consisting of the letters x and o. The letter x represents a positive hybridization, and o represents no hybridization detected for each of the 43 spacer sequences. Panel B is the graphic output from WebLogo. Numbers in each panel represent the spoligotype assay spacer sequences 1–43. Panel C shows the summary graphic of the spoligotypes by collapsing the data into a single row. Legend: x=hybridization observed to spacer, o= no hybridization observed to spacer, ■= positive hybridization in every spoligotype pattern for that individual spacer sequence, □= no hybridization, ●=positive hybridization in >50% of the patterns, ○= no hybridization in >50% of the patterns.

Figure 1 illustrates logo analysis with spoligotyping data. Spoligotyping identified 28 different spoligotypes associated with *M. bovis* from 43 isolates in our collection. The spoligotypes were then coded for sequence logo analysis (Figure 1A). Letter designations were chosen for compatibility with the WebLogo program as described in Methods. The tallest x and o characters represent areas of absolute concordance between the patterns chosen for analysis. Where differences occur, the ratio of those spoligotypes showing hybridization to those that do not is represented by relative height differences of the characters in that column. The resulting spoligologo (Figure 1B) shows that spacers 20, 25, 26, and 38 are present in all 28 spoligotype patterns. The absence of spacers 39–43 in these spoligotype patterns is consistent with an identification of *M. bovis* (13). The greatest polymorphism between the patterns appears in spacers 3 through 16 (Figure 1B). Spoligotype patterns probably evolve through the loss of spacer sequences through a variety of mechanisms (7,20). We try to extrapolate back to the hypothetical founder of these *M. bovis* isolates, which we believe had spacers 1–38 and not 39–43. No isolate with this probable *M. bovis* founder spoligotype has yet been observed in the Wadsworth Center spoligotype database.

**Figure 2:**
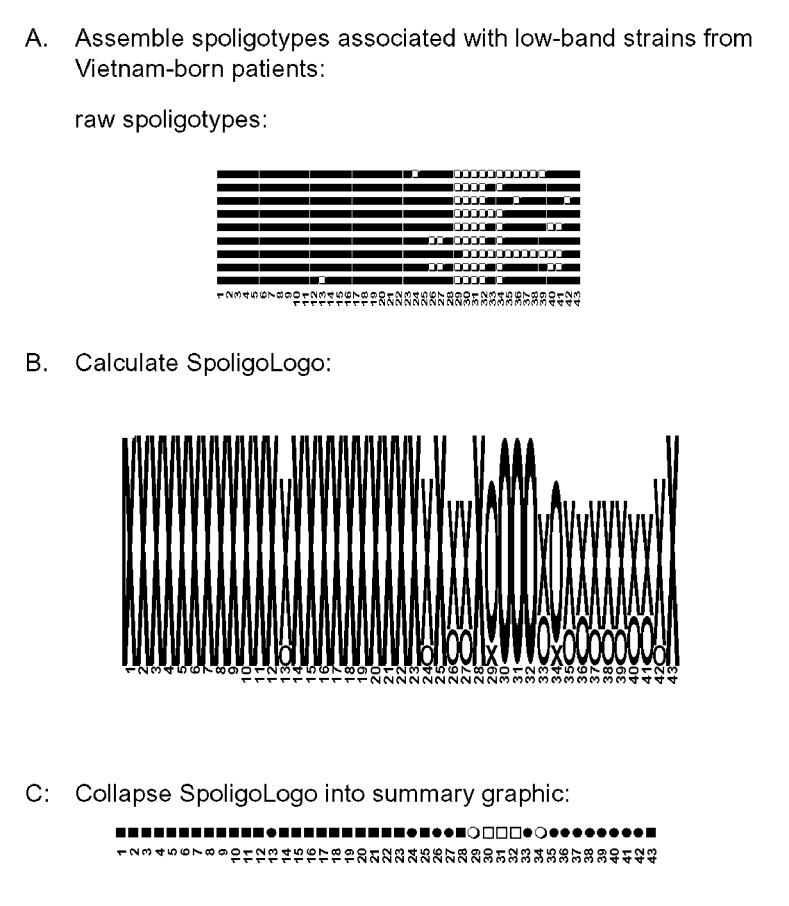
Logo analysis on nine different spoligotypes observed for *Mycobacterium tuberculosis* isolates from Vietnam-born patients in Massachusetts demonstrating fewer than six copies of IS*6110* by RFLP analysis. Legend: x= hybridization observed to spacer, o= no hybridization observed to spacer, ■= positive hybridization in every spoligotype pattern for that individual spacer sequence, □= no hybridization, ●=positive hybridization in >50% of the patterns, ○= no hybridization in >50% of the patterns.

Foreign-born persons, especially those from regions with high TB case rates, are of concern for TB transmission in the United States (21,22). The low-band *M. tuberculosis* strains from Vietnam-born patients, which we selected, produced another spoligologo pattern (Figure 2). The ability of spoligologo analysis to collapse even a small selection of spoligotype patterns from a select group of strains (Figure 2A) into the possible founder spoligotype can be observed in the summary (Figure 2C). However, additional typing methods would be required to verify that the strains are related, rather than exhibiting convergent evolution of their respective spoligotypes.

As previously suggested (5,7,23), *M. tuberculosis* strains that generally contain spacers 33–43 may form a family that is an intermediate lineage between the Beijing (11,12) and non-Beijing *M. tuberculosis* strain families, such as Haarlem (2).

## Discussion

Spoligotyping, microarrays, and DNA-chips are all examples of reverse-hybridization array-based assays. Although microarrays and DNA-chips can contain thousands of bits of data, the principle behind them is similar to that of the spoligotype assay, which uses a simple 1 x 43 array. Array-based assays use reverse hybridization in which a labeled sample is probed against a series of proteins or nucleic acids that are bound to a solid support, such as a nylon membrane or silica. The result for each potential binding event can be recorded as yes or no. The binary nature of array-based assays allows the data to be analyzed usefully with algorithms associated with motif recognition, such as sequence logo analysis. The relative low cost and simplicity of the spoligotype assay means it can be performed by many laboratories and the digital nature of the data facilitates the exchange of information among researchers.

The growth in the availability of array-based assays has outpaced the ability of conventional software analysis packages to provide every possible method of analysis. Customized versions of software are extremely expensive, and researchers, who want to implement these protocols without specialized software, lack methods of collating the large amounts of data. Problems arise when attempts are made to judge the significance of similar but nonidentical array data. Identifying possible families in these array patterns may be important in understanding the evolution and spread of pathogens such as *M. tuberculosis*. Our method for collapsing array-based data can be used to find and present patterns or signature motifs in these types of data.

As previously noted (1), cluster analysis of large RFLP databases is difficult for a combination of reasons, including software failure and intralaboratory variations. Digital data, such as spoligotyping, mycobacterial interspersed repetitive unit (24), and variable number tandem repeat analyses (25), will probably form the basis for any large DNA fingerprinting projects in the future (26).

The design of large genotyping projects should include multiple methods (2,3,10,23). Strains that cluster by one typing method must be analyzed by other methods to ensure that the groupings represent clusters of true relatedness and not cases of convergent evolution.

Bioinformatic analyses, like logo analysis, may prove useful in obtaining further data from the large *M. tuberculosis* complex DNA typing databases already in existence. Spoligologo analysis is a graphic method of presenting similar spoligotypes that may elicit useful insights into the geographic spread of tuberculosis. Potential families of TB strains could be identified on the basis of their logo; these strains could then be analyzed by additional DNA-typing methods to confirm the relationship, followed examining relevant patient data (e.g., country of birth). A similar analysis was performed for the *M. tuberculosis* W-Beijing family (11) that helped elucidate the evolution of a multidrug–resistant strain in New York City. Spoligologo analysis could help identify more of these families, determine their global origin, and evaluate their spread.

Determining the sources and spread of tuberculosis is an important tool in preventing further infections. Understanding the geographic origin of an *M. tuberculosis* DNA fingerprint could be useful, especially in understanding the sources and spread of strains in the U.S. foreign-born population, among whom differentiating recently transmitted disease from reactivation of a past exposure can be difficult.
